# A novel de novo canonical splice site mutation in the PTCH1 gene in a male patient with mild psychomotor retardation and autistic traits: a case report

**DOI:** 10.1038/s41439-023-00254-9

**Published:** 2023-09-26

**Authors:** Parisa Mashayekhi, Mir Davood Omrani, Seyed Hasan Tonekaboni, Ali Dehghanifard

**Affiliations:** 1 Molecular Medicine Department, Biotechnology Research Center, Pasteur Institute of Iran, Tehran, Iran; 2 Urogenital Stem Cell Research Center, Shahid Beheshti University of Medical Sciences, Tehran, Iran; 3 Pediatric Neurology Research Center, Shahid Beheshti University of Medical Sciences, Tehran, Iran

**Keywords:** Development, Autism spectrum disorders

## Abstract

Basal cell nevus syndrome (BCNS), or Gorlin syndrome, is a rare autosomal dominant disorder caused by mutations in the tumor suppressor gene PTCH1 with complete penetrance and variable expressivity characterized by a broad spectrum of developmental anomalies and a predisposition to neoplasms. Herein, we report a novel de novo splice site mutation in the PTCH1 gene related to mild developmental delay and autistic traits in a 4-year-old male patient.

This research received no specific grant from any funding agency in the public, commercial, or not-for-profit sectors.

Basal cell nevus syndrome (BCNS), or Gorlin syndrome (OMIM, 109400), is a rare autosomal dominant disorder characterized by multiple basal cell carcinomas, odontogenic keratocytes, skeletal abnormalities, pits in the palms and soles, unusually large head size (macrocephaly) with a prominent forehead, skeletal abnormalities, and facial dysmorphism. Signs and symptoms are typically present from birth or become apparent in early childhood^1^. This syndrome affects both sexes similarly, with a greater frequency in Caucasians and a lower frequency in Blacks and Asians^2^.

PTCH1 mutations are the main molecular defects related to Gorlin syndrome^3^. PTCH1 encodes a transmembrane glycoprotein that is an essential part of hedgehog (Hh) signaling, which regulates cell differentiation, tissue polarity, and cell proliferation and is a crucial regulator of embryonic development and tumorigenesis. In addition, this gene is a tumor suppressor that stops the uncontrolled proliferation of cells^4^. PTCH1 mutations prevent the production of the Patched-1 protein or result in the production of abnormal receptors that cannot effectively suppress cell growth and division^5^. Thus, cells proliferate uncontrollably and form tumors that are characteristic of Gorlin syndrome. There is no clear explanation for the development of other signs and symptoms related to this condition, such as macrocephaly, skeletal abnormalities, and palmar and plantar pits.

A 4-year-old male patient, the first child of a healthy nonconsanguineous Iranian family, was referred to our clinic because of psychomotor retardation with delayed motor milestones and speech development and autistic traits. He was born through cesarean section at 42 weeks of pregnancy with a weight of 3600 g (>97th centile), length of 55 cm (>97th centile), and occipitofrontal circumference (OFC) of 35.5 cm (>97th centile). Apgar scores were 9 and 10 at 1 and 5 min, respectively. He did not have any history of prenatal complications and/or exposure to teratogens.

On clinical examination, he had a coarse face, mild hypertelorism, epicanthic folds, macrocephaly, frontal bossing, and palmar/plantar pits that were more visible after bathing. He made little eye contact, was interested in things around him, and tended to show limited interest in people.

His brain MRI showed ventricular dilatation. His audiography test, electroretinography (ERG), and visual evoked response (VER) were normal. Chromosome analysis by G-banding at 450-band resolution revealed a normal male. There was no evidence of storage, metabolic, or neurodegenerative disorders in laboratory investigations. In addition, his parents were not related and did not show any abnormal findings in our clinical examinations.

To investigate the underlying genetic factors, peripheral blood was taken from the patient and collected in ethylenediamine tetraacetic acid (EDTA) tubes. DNA was extracted using a GeneAll DNA extraction kit according to the manufacturer’s instructions. For whole-exome sequencing, exome libraries were prepared using the Agilent SureSelect Human All Exon V7 Capture Kit according to the manufacturer’s instructions. Then, the Illumina NovaSeq 6000 platform was used to perform paired-end sequencing. Variants were evaluated based on the available information from multiple databases (including OMIM, HGMD, ClinVar, Exome Sequencing Project, 1000 Genomes, and dbSNP), published literature, clinical correlation, and the predicted functional or splicing impact according to evolutionary conservation analysis and computational tools (including MutationTaster, PolyPhen, and SIFT). The evidence for phenotype-causality was then evaluated for each variant identified by the filtering strategies, and variants were classified according to the ACMG guidelines. We found a novel de novo mutation, NG_007664.1(NM_000264.3):c.654 + 1 G > T, in the splice donor site in intron 4 of the PTCH1 gene that resulted in disruption of RNA splicing. We scored the variant according to the ACMG guidelines and classified it as a pathogenic variant on the basis of the criteria PVS1 (null variant in gene PTCH1), PS2 (negative parental sample test), and PM2 (evidence in the population database). Our result was confirmed by Sanger sequencing using an ABI Prism3500 Genetic Analyzer (Applied Biosystems, Foster City, CA, USA), Fig. 1.

**Fig. 1:**
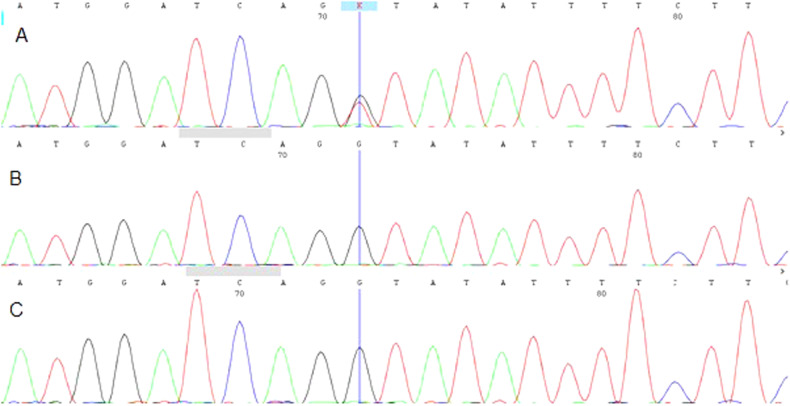
The sanger sequencing results. Electropherogram showed the heterozygous germline point mutation [c.654+1 G > T] in the PTCH1 gene for patient **A** and the homozygous wild-type allele for his father **B** and his mother **C**.

The detected mutation is a novel de novo mutation and is located in the splicing region; de novo PTCH1 mutations are common, affecting up to 50% of patients^6^.

According to the ACMG scoring guidelines, the detected mutation is classified as a pathogenic variant. The identified mutation is consistent with previous reports of splice site mutations causing Gorlin syndrome. Chise Kato et al. discovered 73 mutations in the PTCH1 gene; 10 of them (14%) were putative splicing mutations, and eight of the 10 were located near the splice donor site^7^. Consistent with previous studies identifying intronic splicing mutations, we found no specific clinical features that would have predicted the presence of a splicing mutation.

It should be noted that splicing errors can also be caused by silent mutations, where single-base substitutions do not alter specific amino acids. For example, in a study of patients with neurofibromatosis where mRNA and genomic DNA were compared, it was found that 37% of the patients had splice site mutations that were detected without cDNA sequencing, while 50% of them had mutations that resulted in splicing aberrations and were confirmed by mRNA analysis^8^. Because of the role of splicing site mutations in the pathogenesis of Gorlin syndrome, additional cDNA sequencing after the identification of a genomic mutation, particularly in the case of silent mutations, could help uncover the underlying mechanisms.

As noted in this case, there is some evidence of the occurrence of behavioral, psychiatric, and learning problems in patients with Gorlin syndrome^9^. The sonic hedgehog signaling pathway is involved in embryonic development^10^; as a morphogen, it plays a crucial role in determining the neuron’s identity and assembling differentiated cells in the correct spatial arrangement and proportion^11^. SHH overexpression or inactivation of its target (PTCH1 gene) results in neural tube expansion. In addition, DC Ung et al. showed that Ptchd1 deficiency caused synaptic and cognitive dysfunctions in mice^12^. Considering the role of PTCH1 in embryonic development and neuronal activity, it is not unlikely that the PTCH1 mutation plays a role in the etiology of autism and should be considered in autistic patients.

Consistent with previous studies, we did not find a genotype-phenotype correlation in the current case, which suggests that PTCH1 mutations cannot be used to predict disease burden and improves the understanding of phenotypic variability.

To ensure optimal care of patients with Gorlin syndrome, a multidisciplinary approach is recommended. For early diagnosis of basal cell carcinoma (BCC), a whole-body inspection should be performed every year by a dermatologist; patients should be examined every two years for odontogenic keratocysts (OKCs) of the jaw using orthopantomography (OPG) or magnetic resonance imaging (MRI); MRI should be performed in patients with clinical symptoms or abnormal psychomotor development; and routine developmental screening should be considered for early detection of developmental delay and subsequent intervention or support^13^.

## HGV Database

The relevant data from this Data Report are hosted at the Human Genome Variation Database at 10.6084/m9.figshare.hgv.3330.
